# Inflammation between defense and disease: impact on tissue repair and chronic sickness

**DOI:** 10.15190/d.2015.34

**Published:** 2015-03-31

**Authors:** Elisa A. Liehn, Hector A. Cabrera-Fuentes

**Affiliations:** Institute of Molecular Cardiovascular Research, RWTA Aachen, Aachen, Germany; Institute of Biochemistry, Medical School, Justus-Liebig University, Giessen, Germany; Cardiovascular and Metabolic Disorders Program, Duke-National University of Singapore; National Heart Research Institute Singapore, National Heart Centre Singapore

**Keywords:** inflammation, cardiovascular disease, ischemic heart disease, atherosclerosis, stroke, obesity, diabetes, myophaties, cancer, ionizing radiation

## Introduction

Inflammation represents a complex biological response to harmful stimuli. The word comes from the latin “*inflammatio”, *literally meaning burning or setting on fire-, being mostly considered a negative response of the organism to the infection (pain, heat, redness, swelling and loss of function). However, we are now aware that inflammation is a protective response, involving immune cells, blood vessels, and a wide range of molecular mediators, which must eliminate the initial cause of cell injury, clear out necrotic cells and damaged tissue, initiating tissue repair.

Harmful stimuli are represented not only by microbial invasion, but also by physical- (trauma, injury of all kind), chemical-, mechanical stress, some metabolic disorders, electrolyte imbalance, or absence of vital components (oxygen, glucose). In this context, each organism has different abilities to respond and control the inflammatory reaction, challenging the treatment of such kind of disease and explaining the difficulty to find a unified therapeutic strategy^1^.

## Plauers of inflammatory reaction

Depending on the stimuli, the inflammatory reaction can be rapid and prompt (acute) or persistent (chronic), limited to a specific region (local) or extended to the whole organism (systemic). In all cases, the same components are always involved, however in different proportions: activation of different plasma cascade systems (complement, coagulation, fibrolysis), cell-derived mediators (nitric oxide, lysosome granules, tumor necrosis factor-alpha-α, interleukin 6, interleukin 8, leukotrienes, prostaglandins) induce vascular dilatation, as well as leukocyte activation, migration and extravasation.

The immune cells or leukocytes are the main effectors; they are critically involved in the initiation, controlling and modulation of inflammatory reaction, responding to the soluble factors and mediating the communication between the involved cells. They derive from multipotent stem cells located in the bone marrow and are stored in lymphatic organs (spleen, lymph nodes, thymus), freely patrolling throughout the body and interfering or accumulating every time when the normal physiology of the tissue is disturbed.

Currently, the leukocytes are intensively studied to understand their morphologic and functional heterogeneity and to develop therapeutic strategies to reduce the tissue damage following the inflammatory processes.

Neutrophils are the most abundant circulating immune cells, being primarily involved in the anti-microbial protection. Nevertheless, neutrophils are involved in other inflammatory processes as well, releasing proteolytic enzymes and reactive oxygen species, and directly injuring the surrounding cells. They also release chemotactic factors, inducing monocyte accumulation in injured tissue. Two monocyte populations have been identified: the inflammatory or classical monocytes exhibiting phagocytic and inflammatory functions and clearing the wound of cellular debris, and the resident or nonclassical monocytes, promoting healing and tissue repair. Lymphocytes have many subtypes, and are coordinated by a complex chemokine system, whose roles are still under investigation. They seem to be responsible for the fine-tuning of inflammatory and reparative processes.

Some leukocytes migrate into the tissues becoming permanent residents and have been given specific names (histiocytes, dendritic cells, mast cells, etc.). These cells have aspecific roles in maintaining the homeostasis of the tissue, but become active as immune cells as soon as the inflammatory processes are initiated.

## Resolution of inflammation

In order to have proper healing and repair of the tissue after an injury, it is essential to terminate the inflammatory reaction^2^. While the inflammatory molecules are consumed, downregulated or cleaved by matrix metalloproteinase, a lot of anti-inflammatory mediators are released (such as transforming growth factor-beta, interleukin 10^3^, lipoxins, interleukin-1 antagonists). The pro-inflammatory cells undergo apoptosis (such as neutrophils)^4^, while the survival of the remaining cells increases.

However, on one hand the outcome depends on the tissue in which the injury has occurred and, on the other hand, the outcome also depends on the harmful stimuli that are causing it. This can result in the complete resolution of the injury, with the morphologic and functional restauration of the affected organ, mostly if the inflammatory reaction was short and the organ is capable of regeneration^5^. However, larger tissue destruction results in formation of a fibrotic scar, replacing the destroyed tissue^6^.

Microbial invasion will induce formation of abscesses, containing pus, which normally heal after eliminating their content.

If the body does not succeed to restore the normal homeostasis, or if the stimuli persist over the time, the inflammatory reaction becomes chronic and the effects become systemic, extended to the whole organisms, mostly with irreversible damage.

## Inflammatory diseases

Inflammatory disorders underlie a vast variety of human diseases. If one of the main components of the immune system is disturbed (soluble mediators or cells), the entire inflammatory process will be abnormal, due to the cascade activation of the following factors. Beside the classical inflammatory diseases, a lot of other human pathologies have been shown to be initiated and sustained by the inflammatory processes.

### Classical inflammatory diseases**

This group of diseases is clearly associated with abnormalities of the immune system and a disturbed inflammatory response: autoimmune diseases, chronical infections (glomerulonephritis, prostatitis, sarcoidosis, and vasculitis), rheumatoid arthritis, asthma, transplant rejection, allergies, etc. The treatment of these diseases is mainly directed against the inflammatory reaction and its components.

### Non-immune diseases with etiological origins in inflammatory processes

In the last decades, it has been shown that many other diseases are sustained and modulated by the inflammatory reaction. Tumor growth, atherosclerosis and heart diseases, myopathies, metabolic diseases or even depression seem to progress due to the interference of inflammatory components. Therefore, current studies have analyzed new therapeutic approaches, targeting inflammation to reduce the progression and the side effects of these diseases.

## Obesity

Obesity is now considered the disease of the twentieth century. The accumulation of fat tissue is not only a passive process, but involves all organs and tissues, starting a global reaction, assembling the chronic inflammatory reaction, which was called systemic inflammation^7^. High caloriec intake has been associated with increased inflammatory markers (interleukin 6, interleukin 8, tumor necrosis factor-alpha, leptin, etc)^8,9^.

## Diabetes

Hyperglycemia induces interleukin 6, but also other inflammatory cytokines production, mostly from endothelial cells and macrophages^8,10^, increasing the systemic inflammation and predisposing to chronical tissue damages.

## Myopathies

Exercise induces acute inflammatory reaction in muscle cells, forcing the muscle growth^11^, while regular and moderate physical activity seems to decreases the inflammation^12^.

## Cancer

Neagu et al. described in this issue the fine balance between inflammation and tumorigenesis. Inflammation is able to induce but also to sustain malignant transformation and growth. It sustains the proliferation and survival of tumor cells and contributes to their spreading (metastasis)^13^. Many studies reveal the molecular pathways linking the cancer with inflammatory processes^14-16^. Therefore, new therapeutic strategies targeting/modulating the inflammation and related-molecules are proved in treating the all phases of malignant transformation (initiation, growth and metastasis).

## Atherosclerosis

Atherosclerosis represents the thickening of the vascular wall due to the accumulation of cholesterol and leukocytes^17^. Many years was thought that there is a passive accumulation of cholesterol inside of the vascular wall, therefore the main therapeutic strategies had involved the reduction of fat in circulating blood. Nevertheless, the interference of the inflammation seems to be more relevant in inducing, sustaining and progression of atherosclerosis, and therefore, targeting immune cells and mediators represents a viable therapeutic strategy for the future, as propose Kubo et al. in this current issue.

## Ischemic heart disease

Myocardial infarction represents the death of cardiac cells due to the lack of oxygen following the dangerous narrowing of an atherosclerotic vessel. Dying cardiac cells release danger signals and activate inflammatory reaction, as described by Frangogiannis et al. in the current issue. The inflammatory reaction is responsible for cleaning the dead cell debris, but also to initiate healing and scar formation^18^. Targeting the inflammatory reaction in this context, seems to have catastrophic results^19^. Therefore Frangogiannis et al. propose a specific intervention on interleukin-1 family members, reducing the side effects on repair and remodeling of the infarcted heart.

## Neurological diseases

Neurological diseases induce temporary or permanent disabilities associated with huge economic losses. The therapeutic possibilities evolved over the years; however, there are still unacceptable limited. New mechanistic insights have shown a possible link between these type of disorders and inflammatory reaction. Thus, negative cognition (stress, violence or deprivation) is able to trigger an up-regulation of systemic inflammatory reaction, leading to depression^20^. Cerebrovascular diseases are modulated and controlled by inflammatory system, similar to ischemic heart diseases, as Uzoni et al. summarized in the current issue.

## Ionizing radiation

Ionizing radiation becomes more and more known, due to the environmental changes, but also due to the increased terrorism threat or the possibility of a nuclear incident. The effects of ionizing radiation are deleterious and mainly irreversible, leading to significant morbidity and mortality. The current therapeutic solutions are limited; however, the knowledge about the mechanisms involved is increasing, as reviewed by Singh et al. in this issue. Ionizing radiation activates the inflammatory-related mechanisms, such as the mitogen-activated protein kinase (MAPK), phosphoinositide 3-kinases (PI3K), Nuclear factor (NF)-κB, etc, which can eventually be targeted to assure the protection and to increase the survival.

## Conclusion

Summarizing the current knowledge, the inflammation includes various factors and cells responsible to sustain the structure and function of all tissues and organs assisting during the pathological events and repair. Chronic presence of the inflammatory mediators due to different causes results in permanent damages, with severe consequences. The therapeutic strategies targeting the inflammatory mediators proved to be a real and efficient tool to treat a broad variety of other diseases, in addition to the known classical inflammatory pathologies.

## References

[1] Frangogiannis Nikolaos G (2006). Targeting the inflammatory response in healing myocardial infarcts.. Current medicinal chemistry.

[2] Serhan Charles N, Savill John (2005). Resolution of inflammation: the beginning programs the end.. Nature immunology.

[3] Sato Y, Ohshima T, Kondo T (1999). Regulatory role of endogenous interleukin-10 in cutaneous inflammatory response of murine wound healing.. Biochemical and biophysical research communications.

[4] Greenhalgh D G (1998). The role of apoptosis in wound healing.. The international journal of biochemistry & cell biology.

[5] Serhan Charles N (2008). Controlling the resolution of acute inflammation: a new genus of dual anti-inflammatory and proresolving mediators.. Journal of periodontology.

[6] Dobaczewski Marcin, Gonzalez-Quesada Carlos, Frangogiannis Nikolaos G (2010). The extracellular matrix as a modulator of the inflammatory and reparative response following myocardial infarction.. Journal of molecular and cellular cardiology.

[7] Kershaw Erin E., Flier Jeffrey S. (2004). Adipose Tissue as an Endocrine Organ. The Journal of Clinical Endocrinology & Metabolism.

[8] Esposito K. (2002). Inflammatory Cytokine Concentrations Are Acutely Increased by Hyperglycemia in Humans: Role of Oxidative Stress. Circulation.

[9] van Dijk Susan J, Feskens Edith J M, Bos Marieke B, Hoelen Dianne W M, Heijligenberg Rik, Bromhaar Mechteld Grootte, de Groot Lisette C P G M, de Vries Jeanne H M, Müller Michael, Afman Lydia A (2009). A saturated fatty acid-rich diet induces an obesity-linked proinflammatory gene expression profile in adipose tissue of subjects at risk of metabolic syndrome.. The American journal of clinical nutrition.

[10] Shoelson Steven E, Lee Jongsoon, Goldfine Allison B (2006). Inflammation and insulin resistance.. The Journal of clinical investigation.

[11] Toth Michael J, Matthews Dwight E, Tracy Russell P, Previs Michael J (2005). Age-related differences in skeletal muscle protein synthesis: relation to markers of immune activation.. American journal of physiology. Endocrinology and metabolism.

[12] Smith J K, Dykes R, Douglas J E, Krishnaswamy G, Berk S (1999). Long-term exercise and atherogenic activity of blood mononuclear cells in persons at risk of developing ischemic heart disease.. JAMA.

[13] Mantovani Alberto, Allavena Paola, Sica Antonio, Balkwill Frances (2008). Cancer-related inflammation.. Nature.

[14] Balkwill Frances R, Mantovani Alberto (2012). Cancer-related inflammation: common themes and therapeutic opportunities.. Seminars in cancer biology.

[15] Del Prete Annalisa, Allavena Paola, Santoro Giuseppe, Fumarulo Ruggiero, Corsi Massimiliano M, Mantovani Alberto (2011). Molecular pathways in cancer-related inflammation.. Biochemia medica.

[16] Locatelli Marco, Ferrero Stefano, Martinelli Boneschi Filippo, Boiocchi Leonardo, Zavanone Mario, Maria Gaini Sergio, Bello Lorenzo, Valentino Sonia, Barbati Elisa, Nebuloni Manuela, Mantovani Alberto, Garlanda Cecilia (2013). The long pentraxin PTX3 as a correlate of cancer-related inflammation and prognosis of malignancy in gliomas.. Journal of neuroimmunology.

[17] Liehn Elisa A, Zernecke Alma, Postea Otilia, Weber Christian (2006). Chemokines: inflammatory mediators of atherosclerosis.. Archives of physiology and biochemistry.

[18] Liehn Elisa A, Postea Otilia, Curaj Adelina, Marx Nikolaus (2011). Repair after myocardial infarction, between fantasy and reality: the role of chemokines.. Journal of the American College of Cardiology.

[19] Roberts R, DeMello V, Sobel B E (1976). Deleterious effects of methylprednisolone in patients with myocardial infarction.. Circulation.

[20] Berk Michael, Williams Lana J, Jacka Felice N, O'Neil Adrienne, Pasco Julie A, Moylan Steven, Allen Nicholas B, Stuart Amanda L, Hayley Amie C, Byrne Michelle L, Maes Michael (2013). So depression is an inflammatory disease, but where does the inflammation come from?. BMC medicine.

